# The efficacy of laparoscopy in the diagnosis and management of chronic abdominal pain

**DOI:** 10.4103/0972-9941.72594

**Published:** 2010

**Authors:** Gouda M El-labban, Emad N Hokkam

**Affiliations:** Department of General Surgery, Faculty of Medicine, Suez Canal University, Ismailia, Egypt

**Keywords:** Abdominal pain, laparoscopy, minimally invasive

## Abstract

**BACKGROUND:**

Chronic abdominal pain is a difficult complaint. It leads to evident suffering and disability, both physically and psychologically. Many diagnostic and therapeutic procedures have been described in literature, but with little proof or evidence of success. Laparoscopy is one of the modalities that could be of benefit in such cases. We aim to evaluate the diagnostic and therapeutic value of laparoscopy in cases with chronic abdominal pain.

**MATERIALS AND METHODS:**

Thirty patients with chronic abdominal pain were included in this prospective descriptive cross-sectional study. The pain in all patients was of unclear etiology despite all the investigative procedures. All patients were subjected to laparoscopic evaluation for their conditions. The findings and outcomes of the laparoscopy were recorded and analyzed.

**RESULTS:**

The most common site of pain was the periumbilical region (30%). A definitive diagnosis was made in 25 patients (83.3%), while five patients (16.7%) had no obvious pathology. Adhesions were the most common laparoscopic findings (63.3%) followed by appendiceal pathology (10%), hernia (3.3%), gall bladder pathology (3.3%), and mesenteric lymphadenopathy (3.3%). Postoperatively, pain relief was achieved in 24 patients (80%) after two months.

**CONCLUSION:**

Laparoscopy is an effective diagnostic and therapeutic modality in the management of patients with chronic abdominal pain.

## INTRODUCTION

Chronic abdominal pain is a common disorder both in general practice and in hospitals. Although patients with this type of pain may have undergone numerous diagnostic workups, including surgery, their pain remains a challenge to all known diagnostic and treatment methods. After all, more than 40% of the patients presenting with chronic abdominal pain had no specific etiological diagnosis at the end of their diagnostic workup.[1–4] Chronic abdominal pain is associated with poor quality of life[5] and significant levels of depressive symptoms.[6] Much is known about the prevalence, societal burden, and suffering associated with chronic abdominal pain.[1] Many common organic and functional diseases can cause it. The most common organic conditions include intestinal adhesions,[7,8] biliary causes,[9,10] and appendicular causes,[11] while functional conditions include irritable bowel disease,[12] functional dyspepsia,[13] and various motility disorders.[14] Abdominal wall pain is also common and frequently mistaken for visceral pain.[15,16] After ruling out common diseases by careful investigations, many patients are still undiagnosed and represent a major diagnostic challenge to the surgeon.[17] With the introduction of laparoscopic surgery, a new tool has been added to our knowledge. The use of this new technology in the diagnosis and management of chronic abdominal pain has been tried in previous studies.[18–20] Laparoscopy can identify abnormal findings and improve the outcome in a majority of patients with chronic abdominal pain, as it allows surgeons to see and treat many abdominal conditions that cannot be diagnosed otherwise.[4,19] It is a safe and effective tool and can establish the etiology and allows for appropriate interventions in such cases.[21] Abdominal adhesions are the most likely findings, especially in patients with a past history of abdominal operations.[22] Other findings such as appendiceal pathology, hepatobiliary causes, and endometriosis can be discovered and dealt with.[18] However, the role of laparoscopy in chronic abdominal pain is still debated by some authors who deny its value in adhesiolysis and consider it controversial and not evidence-based, and therefore, do not recommend it as a treatment for adhesions in patients with chronic abdominal pain.[23,24] In the present study we aim to evaluate the use of the laparoscope in the diagnosis and management of patients with chronic abdominal pain.

## MATERIALS AND METHODS

Between February 2008 and January 2009, a total number of 30 consecutive patients with chronic abdominal pain were enrolled in this prospective descriptive cross-sectional study. They were recruited from the Outpatient Clinic of the Surgery Department, Suez Canal University Hospital. Ismailia, Egypt. After approval of our Ethics Committee, all the patients underwent laparoscopic surgery for evaluation and management of their chronic abdominal pain. We defined chronic abdominal pain as a continuous or intermittent abdominal pain with daily intake of analgesics, and a duration of at least three months.[25,26]

In all the patients, the pain was of unclear etiology, despite physical, laboratory, and radiographic evaluation. The patients who presented with acute abdominal pain were excluded from the study. Also patients with known abdominal malignancy, patients being treated by psychiatrists, and patients under the age of 18 years were excluded.

All of the studied patients were subjected to a complete preoperative evaluation through a medical history and an abdominal examination to find out if there were any organic diseases of the alimentary tract or the abdomen. Special concern was given to any past history of abdominal operations. Associated symptomatology, such as vomiting, fever or abdominal distention, were noted and recorded. Routine preoperative laboratory investigations including coagulation profile and complete blood count were performed. A total of 57 imaging studies (excluding plain films) had been done before laparoscopy, including 27 abdominal ultrasounds, 15 computed tomographies, (CT), 10 barium enemas, and five upper gastrointestinal endoscopies

### Operative technique

The procedure was entirely performed with the patient under general anesthesia. If there was a previous upper midline incision or massive intra-abdominal adhesions were suspected, the Veress needle was passed through the abdominal wall in an area with no scars, most often in the left upper quadrant of the abdomen, a few centimeters below the costal margin. After establishment of the pneumoperitoneum, a standard three-trocar technique was used (10-mm optic via umbilical trocar and two 5-mm lateral trocars). A fourth 5-mm trocar was inserted in a few cases. The whole abdominal cavity was inspected carefully starting from the liver, gallbladder, anterior surface of the stomach and spleen. With fine smooth graspers, these structures could be touched safely and elevated for further inspection. The small bowel was examined using these atraumatic graspers. It was inspected thoroughly from the ligament of Treitz to the ileocaecal valve, keeping in mind the fact that the loops with the large bit had to be grasped as much as possible to avoid the pinpoint fixation of the bowel with its perforation risk. The colon including the appendix was inspected in the same manner as the small bowel. Finally, the gynecological organs and peritoneal surfaces were inspected. If adhesions were seen between the intestinal loops and the abdominal wall or between the abdominal organs, they were dissected with a scissors in a vast majority of patients. Electrocautery was used mainly for hemostasis and as a dissection technique in few cases. The dissection was made close to the abdominal wall to avoid injury to the bowel loops. Other laparoscopic procedures such as appendectomy, cholecystectomy, hernia repair, and biopsies were performed according to the patient’s condition.

### Postoperative evaluation

After the laparoscopy, postoperative hospital stay was recorded. Standard diclofenac sodium 75 mg was used for postoperative pain relief. All the patients were re-evaluated after two months, six months, and one year. The pain in the late postoperative period was classified into: worse, unchanged, less pain, and disappearance of pain. Less pain and disappearance of pain were referred to as positive outcomes, while unchanged and worse pains were referred to as negative outcomes.

### Statistical analysis

Gathered data were processed using the SPSS version 15 (SPSS Inc., Chicago, IL, USA). A Student *t* test was used to test the significance of difference for quantitative variables, while Chi Square and Fisher’s exact tests were used to test the significance of difference for qualitative variables. A probability value (*P*-value) < 0.05 was considered statistically significant.

## RESULTS

The studied patients were in the age group ranging from 20 – 68 years, with a mean age of 36 years. More than half of the patients studied were females (60%). The mean duration of pain was nine months with the range of duration from three to fifteen months. The most common site of pain was the periumbilical region (30%) followed by the right lower abdominal quadrant (23.3%). Twenty patients were using either non-steroidal drugs or pain killers to relieve the pain, and six patients were using proton pump inhibitors. Seventeen patients (56.7%) had undergone at least one previous surgical abdominal procedure. All patient characteristics are summarised in Table 1.

**Table 1:** Baseline characteristics of the studied patients

Characters	Value
Age (years) mean (range)	36 (20-68)
Gender male	12 (40%)
Female	18 (60)
Body mass index	29.3(18-41)
Duration of pain (months) Mean (range)	9(3-15)
Site of pain	
Right lower quadrant	7 (23.3%)
Right upper quadrant	6 (20%)
Left lower quadrant	3 (10%)
Left upper quadrant	5 (16.7%)
Periumbilical	9 (30%)
History of previous abdominal surgery	17 (56.7%)
Appendectomy	9
Colecyctemy	5
Spleectomy	2
Hysterectomy	1

The average length of the operative time was 58.7 minutes with the range from 30 to 120 minutes. There were no cases converted to open procedures. Out the 30 patients with chronic abdominal pain, a definitive diagnosis was established in 25 patients (83.3%), while no identifiable cause could be reached in five patients (16.7%).

The most common laparoscopic findings were adhesions (63.3%). Other findings included appendiceal pathology (10%), hernia (3.3%), gall bladder pathology (3.3%), and mesenteric lymphadenopathy (3.3%). All patients with adhesions had undergone previous abdominal surgery, except for two patients who had local adhesions of unknown origin. Three patients showed appendiceal pathology; one of them showed adhesions from the appendix to the adjacent structures and the other two showed thickened appendix and their pathology revealed evidence of chronic appendicitis. Other pathological diagnoses such as small ventral hernia, chronic acalcular cholecystitis, and multiple enlarged mesenteric lymph nodes were found in one patient. Table 2 summarises the laparoscopic diagnoses assigned to all patients.

**Table 2:** Laparoscopic findings, intraoperative data, and postoperative characteristics

Findings		Value
Duration of operation (minutes)		58.7 ± 14.1
Mean ± SD (range)		(30 – 120)
Laparoscopic findings	Adhesions	(63.3%)
	Hernia	(3.3%)
	Abnormal appendix	(10%)
	Abnormal gall bladder	(3.3%)
	Enlarged lymph node	(3.3%)
	Normal	(16.7%)
Postoperative complications	None	(83.3%)
	Bleeding	(6.7%)
	Infection	(10%)
Postoperative hospital stay (days)	Mean (Range)	3.6 (2 – 9)

Laparoscopic management included adhesiolysis (19), appendectomy (3), hernia repair (1), cholecystectomy (1), and lymph node biopsy (1). Five patients had no interventions performed.

Postoperative hospital stay ranged from two to nine days with a mean of 3.6 days.

In most cases no postoperative complications had been reported except in five cases (two cases showed bleeding and three cases showed infection). The bleeding could be dealt with through electrocautary and postoperative transfusion of packed cells with no necessity for laparotomy, while the wound infection responded well to oral antibiotic and daily dressing.

During the time of follow up, all patients were re-evaluated for pain. After two months, positive outcome (less pain or disappearance of pain) was achieved in 24 patients (80%), in 21 patients (70%), after six months, and in 19 patients (63%) after one year; while negative outcome (unchanged or worse pain) was noted in six patients (20%) in the first two months, in nine patients (30%) after six months, and in 11 patients (27%) after one year [Table 3].

**Table 3:** Postoperative pain relief

Duration (months)	Positive outcome (%)	Negative outcome (%)
After 2	80	20
After 6	70	30
After 12	63	27

## DISCUSSION

Chronic idiopathic pain syndromes are among the most challenging and demanding conditions to treat across the whole age spectrum. Potentially it can be unrewarding for both the patients and the medical team.[27] Studies conducted with large community samples or hospital populations imply chronic abdominal pain is a pervasive problem. Abdominal pain was the third most common pain complaint of individuals enrolled in a large health maintenance organisation.[28]

All patients included in the study had chronic abdominal pain, and they were subjected to laparoscopic evaluation after exclusion of all organic causes of the pain by radiographic and laboratory tests. Also when abdominal wall pain was suspected, injections of bubivacaine were administered into the trigger-points in 12 patients. The study confirmed that in this difficult patient group, laparoscopy could safely identify abnormal findings and can improve the outcome in a majority of cases.

A majority of patients had undergone previous abdominal surgery, and not surprisingly, in a majority, adhesions were found. However, a significant number were found to have a variety of other conditions to which their pain could be attributed, while a less number were found to have no clear pathology, during laparoscopy. The overall outcome in this series was positive; most of the patients found significant relief from their chronic pain, postoperatively.

The use of laparoscopy in patients with ill-defined chronic abdominal pain remains controversial.[19] While we and others[4,18,26] have found that most patients with chronic abdominal pain had intra-abdominal adhesions and they responded well to laparoscopic adhesiolysis, Ikard[23] has questioned whether laparoscopic adhesiolysis was beneficial and has suggested that it may not be safe. He stated that adhesions do not cause pain unless they are obstructing and in such cases; the laparoscopic approach cannot provide adequate exposure to the abdomen and may be dangerous.

Whether laparoscopic adhesiolysis is preferable to laparotomy or not is a matter of debate. Some authors[19] believe that adhesions can be elusive to even the most sophisticated imaging studies, while others[29] state that the laparoscopic approach for adhesiolysis is safe, feasible, and offers the advantages of decreased length of stay, faster return to full activity, and decreased morbidity. This debate is also evident in the experimental studies, where Luciano *et al*.[30] have found laparoscopic adhesiolysis effective and associated it with a lesser extent of adhesion recurrence, while Prushik *et al*.[31] have found that open adhesiolysis is more beneficial in minimising adhesion reformation.

Unlike Salky and Edye,[18] we have found a low incidence of chronic appendicitis in this study. It may be attributed to the different selection criteria of the patients and the different definition of chronic abdominal pain in both studies. However, many other studies[4,19,21] agree with us in the low percentage of chronic appendicitis as a cause for chronic abdominal pain.

We found that in a selected patient group, laparoscopic evaluation of chronic abdominal pain is usually associated with a positive outcome (80%) in terms of less or no pain, after two months of laparoscopy, and in 70 and 63% of the patients, after six months and one year, respectively. This finding is justified in many previous studies,[18,21,32] however, the role of laparoscopy from the therapeutic point of view is still ignored by some authors, especially its role in adhesiolysis.[23,24]

In conclusion, laparoscopy has an effective diagnostic role in evaluating patients with chronic abdominal pain, in whom conventional methods of investigations have failed to elicit a certain cause. The therapeutic value of laparoscopy is also accepted and appreciated.
